# Natural Marine Products: Anti-Colorectal Cancer In Vitro and In Vivo

**DOI:** 10.3390/md20060349

**Published:** 2022-05-25

**Authors:** Ningning Han, Jianjiang Li, Xia Li

**Affiliations:** 1 Marine College, Shandong University, Weihai 264209, China; hanningning@mail.sdu.edu.cn (N.H.); lijianjiang@mail.sdu.edu.cn (J.L.); 2 School of Pharmaceutical Sciences, Shandong University, Jinan 250012, China

**Keywords:** natural marine products, colorectal cancer, chemical structures, anti-tumor mechanism

## Abstract

Colorectal cancer, a malignant tumor with high mortality, has a poor prognosis due to drug resistance and toxicity in clinical surgery and chemotherapy. Thus, finding safer and more efficient drugs for clinical trials is vital and urgent. Natural marine compounds, with rich resources and original chemical structures, are applied widely in anticancer treatments. We provide a systematic overview of recently reported marine compounds such as alkaloids, peptides, terpenoids, polysaccharides, and carotenoids from in vitro, in vivo, and clinical studies. The in vitro studies summarized the marine origins and pharmacological mechanisms, including anti-proliferation, anti-angiogenesis, anti-migration, anti-invasion, the acceleration of cycle arrest, and the promotion of tumor apoptosis, of various compounds. The in vivo studies outlined the antitumor effects of marine compounds on colorectal cancer model mice and evaluated their efficacy in terms of tumor inhibition, hepatotoxicity, and nephrotoxicity. The clinical studies summarized the major chemical classifications and targets of action of the clinical drugs that have entered clinical approval and completed approval for marine anticancer. In summary, we present the current situation regarding the application of natural anti-colorectal cancer marine compounds and prospects for their clinical application.

## 1. Introduction

Colorectal cancer (CRC) is the second most common cause of death from cancer in the United States [1]. In the world, CRC ranks third in terms of incidence, but second in terms of mortality. More than 1.9 million new CRC (including anus) cases and 935,000 deaths were estimated to have occurred in 2020, representing about one in ten cancer cases and deaths [2]. The majority of patients with stage I and II colon cancer undergo colectomy without chemotherapy (84%), whereas approximately two-thirds of patients with stage III of the disease (as well as some patients with stage II disease) receive adjuvant chemotherapy to lower their risk of recurrence. These treatments are often accompanied by periodic or chronic diarrhoea, with intestinal dysfunction, including increased stool frequency, urinary incontinence, radiation proctitis, and perianal irritation [3]. Thus, finding a safe and efficient drug for clinical trials is vital and urgent.

Natural marine products have broad chemical diversity and have served as candidates for pharmacological research since 1960 due to their unique biofunctional properties. These compounds are isolated from marine microorganisms and phytoplankton, green, brown, and red algae, sponges, cnidarians, bryozoans, mollusks, tunicates, echinoderms, mangroves, and other intertidal plants and microorganisms. In the last five years, there has been a continuous increase in the study of marine fungi compared with a decrease in new compounds reported from sponges, cnidarians, and bacteria [4]. Aanticancer compounds are mainly derived from alkaloids, polysaccharides, peptides, carotenoids, and terpenoids isolated from marine organisms. As of April 2022, a total of 11 anticancer drugs have been approved by the U.S. Food and Drug Administration (FDA) [5], further demonstrating the great potential of the marine environment as a natural treasure trove for compounds with anticancer activity. This review focuses on natural marine products that have been shown to have a good effect against CRC. The search was performed in PubMed, Web of Science, and Scopus over a 5-year period between 2017 and 2022.

## 2. In Vitro Study of Natural Marine Products against CRC

### 2.1. Alkaloid

**Figure 1:**
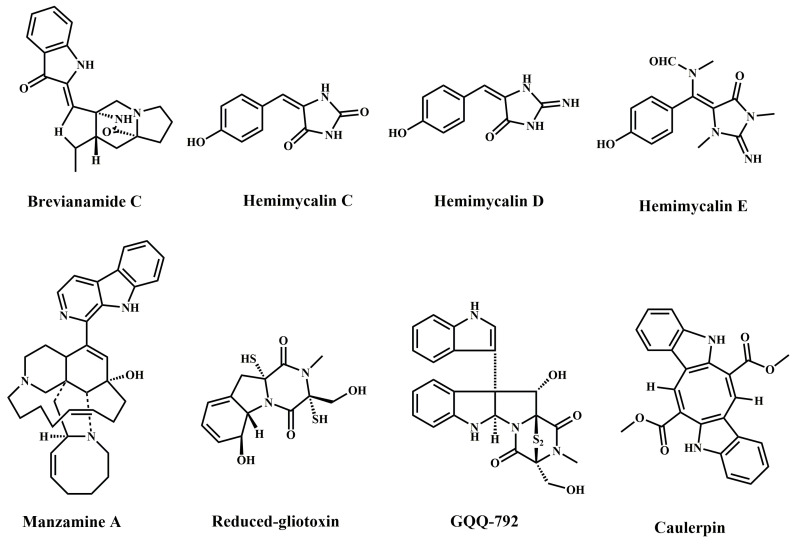
Chemical structures of marine-derived alkaloids against CRC.

Alkaloids are important natural products and are widely distributed in different biological sources, including cyanobacteria, fungi, sponges, sea squirts, actinomycetes, and so on. Of 800 compounds extracted from cyanobacteria, 300 were alkaloids [6,7], probably because cyanobacteria have a significant nitrogen fixation capacity that facilitates alkaloid production. Brevianamide C and reduced-gliotoxin (see Table 1) are both alkaloids of fungal origin [8,9] and facilitate alkaloid production due to their extreme growth conditions and their ability to undergo symbiosis with other marine organisms, such as actinomycetes. Hemimycalin C, D, E, and manzamine A (Table 1) are all sponge-derived alkaloids [10,11], probably due to inhabiting brackish water and their ability to have symbiotic relationships with other marine microorganisms, such as bacteria and fungi, thus facilitating alkaloid production. Trabectedin and lurbinectedin are two alkaloid compounds of marine origin approved by the FDA derived from the sea squirt; thus, the ascidian sea squirt is considered to be a major source of compounds for the treatment of cancer [5]. Unlike other sources, sponge alkaloids such as manzanine A (Table 1) have been shown to inhibit cell proliferation and regulate programmed cell death, such as apoptosis, by targeting topoisomerases and microtubule protein aggregation, and have even been shown to inhibit cell migration and invasion [11]. Caulerpin, a bisindole alkaloid isolated from algae, has been shown to activate AMPK to disrupt glucose metabolism in colorectal cells, leading to cell death [12]. Meanwhile, the immunostimulatory activity of caulerpin is based on the regulation of macrophages via the decreasing of the production of the pro-inflammatory cytokines tumor necrosis factor-α (TNF-α) and interleukin 6 (IL-6) in response to lipopolysaccharide (LPS)-stimulated macrophage RAW 246.7 cells, resulting in anti-inflammatory effects [13]. Anoikis is an intrinsic cellular mechanism that clears detached epithelial cells to maintain tissue homeostasis and development. The catabolism of anoikis contributes to the development of multiple cancers, mainly breast and colon cancers [14]. Enhancing the response of cancer cells to anoikis is therefore considered to be an effective strategy for the treatment of metastatic CRC. In addition, the potency of diketopiperazine alkaloids, such as GQQ-792 and reduced-gliotoxins as well as plinabulin, may be due to sulphide groups, which form mixed disulphides with proteins or antioxidants (e.g., glutathione) and thus have antioxidant effects [5,9,15].The structures of marine-derived alkaloid anti-colorectal cancer compounds are shown in Figure 1.

### 2.2. Peptides

**Figure 2:**
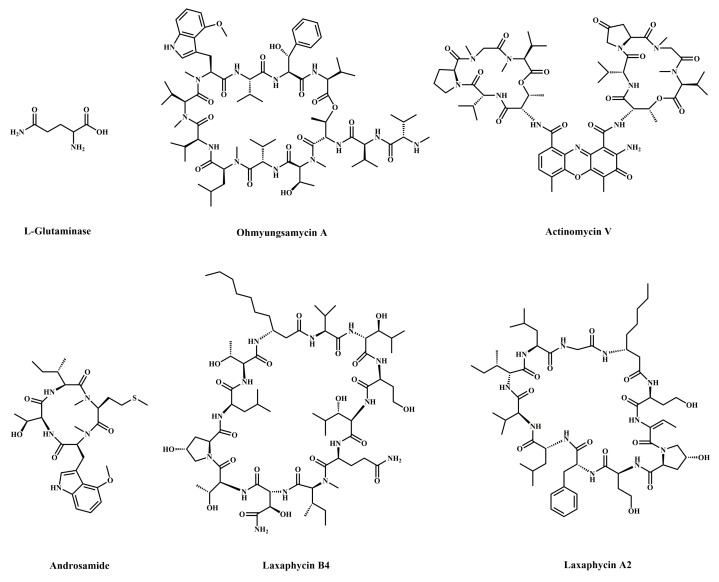
Chemical structures of marine-derived peptides against CRC.

Peptides are amphoteric compounds containing carboxyl and amino groups, which are dehydrated from amino acids, and are commonly found in marine organisms, such as bacteria, tunicates, mollusks, and sponges, among others. For example, L-glutaminase, ohmyungsamycin A, actinomycin V, androsamide, laxaphycin B4, and laxaphycin A2 (Table 2) are of bacterial origin [17,18,19,20,21], the FDA-approved dipeptide plitidepsin is of tunicates origin, and the peptide P6 (Table 2) is of mollusk origin [5,22]. Peptides can be divided into short linear peptides, long linear peptides, cyclic peptides, bicyclic peptides, thiopeptides, tetrapeptides, and so on, based on their chemical structure. They have many biological activities, such as defense, immunity, antibacterial, anti-inflammatory, antioxidant, anti-tumor, and so on, and are important signal transduction and regulation molecules. In recent years, nearly 100 peptides with anti-tumor activity have been discovered in marine organisms, of which more than 90% trigger apoptosis through targeted apoptotic mechanisms involving the mitochondria and death receptor pathways. Both apoptotic pathways require the mitochondrial-mediated activation of caspases. The activation of mitochondrial apoptosis pathways is primarily due to several factors, including reactive oxygen species (ROS) production and Ca^2+^ overload in the cells. When Ca^2+^ homeostasis is disrupted in tumor cells, high levels of ROS are produced, resulting in the activation of cell apoptosis [23]. P6, a novel peptide identified from the marine mollusk *Arca inflata*, was effective at suppressing CRC proliferation by inducing mitochondrial apoptosis. P6 showed a profound anti-tumor effect and induced tumor cell apoptosis by activating the p38 MAPK signaling pathway, promoting ROS production and intracellular Ca^2+^ overload in CRC cells [22]. L-glutaminase was shown to drive early and late apoptosis in LS-174-T and HCT-116 tumor cells, as demonstrated by the acridine orange/ethidium bromide (AO/EtBr) double staining assay [17]. Ohmyungsamycin A, a novel cyclic peptide identified from a marine *Streptomyces* sp., inhibited the proliferation and tumor growth of HCT-116 human CRC cells and induced apoptosis also associated with the modulation of caspase family proteins, in addition to inducing G0/G1 phase cell cycle arrest [18]. Actinomycin V induced apoptosis in CRC cells via mitochondrial and PI3K/AKT pathways, with this being characterized by mitochondrial dysfunction manifested by the loss of mitochondrial membrane potential (MMP) and cytochrome C release, which then activated cleaved caspase-9, cleaved caspase-3, and cleaved poly ADP-ribose polymerase (PARP); however, this apoptotic trend could be reversed by the caspase inhibitor Z-VAD-FMK [19]. The structures of natural marine peptide anti-colorectal cancer compounds are shown in Figure 2.

### 2.3. Terpenes

**Figure 3:**
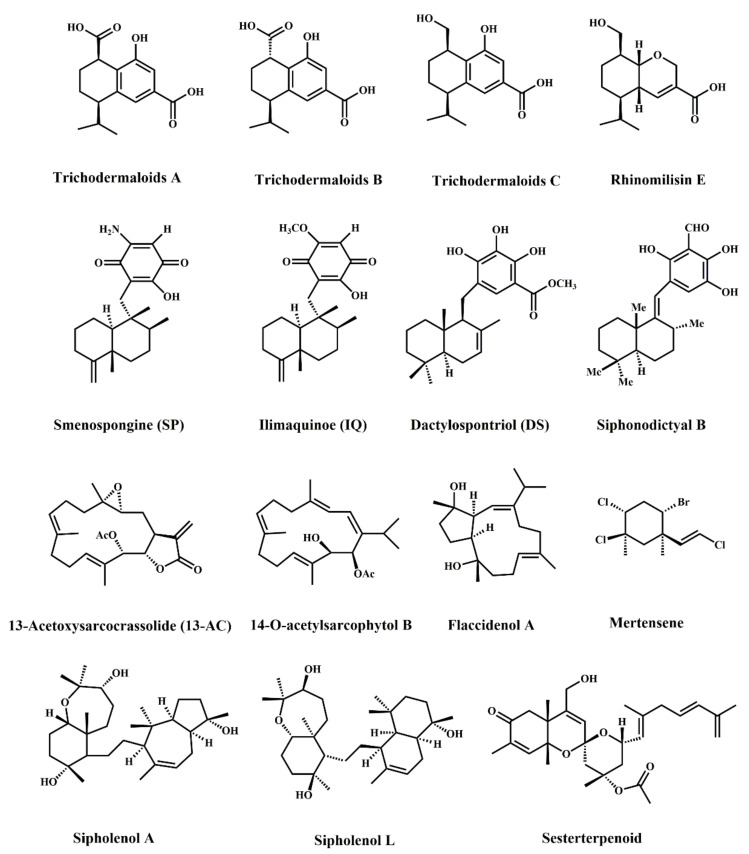
Chemical structures of marine-derived terpenes against CRC.

Terpenes are olefin compounds containing isoprene and can be divided into monoterpenes, sesquiterpenes, diterpenes, triterpenes, terpenes, and polyterpenes according to the number of isoprenes. In Table 3, mertensene is a monoterpene [25]; trichodermaloids A, B, and C, and rhinomilisin E are sesquiterpenes [26]; smenospongine, ilimaquinone, dactylospontriol, 13-acetoxyarcocrassolide siphonodictyal B, flaccidenol A, and 14-O-acetylsarcophytol B are diterpenes [27,28,29,30]; and sipholenol A, sipholenol L, and sesterterpenoid are triterpenes [31,32]; among them, the most common diterpenoids are cyclic diterpenes, as shown in Figure 3. Terpenoids can be derived through the oxidation, rearrangement, and other kinds of chemical structural modification of a variety of alcohols, aldehydes, ketones, carboxylic acids, esters, and other terpenoid structures, which are collectively referred to, in a broad sense, as terpenes. These compounds widely exist in sponges, coral, brown algae, and red algae and have anti-tumor, antiviral, antibacterial, antioxidant, anti-inflammatory, immunomodulatory, antimalarial, anti-parasitic, liver protection, cardiovascular protection, and other biological activities. Terpenoids inhibit tumor cell proliferation by inducing apoptosis and blocking the proliferation cycle of tumor cells, as well as by having anti-tumor cell invasion and metastasis effects, inhibiting tumor angiogenesis, etc.

Three natural terpenoids, smenospongine, ilimaquinone, and dactylospontriol, were isolated from sponges and caused DNA damage and triggered cell death, i.e., so-called mitochondrial apoptosis [27]. Siphonal A and siphonal L increased the nuclear expression of the pro-apoptotic protein-cleaved caspase-3, which effectively drives HCT-116 cellular apoptosis via a caspase-3-dependent pathway. Furthermore, these two compounds induced G2/M and S phase cell cycle arrest, with a concomitant increase in the pre-G cell population, indicating a possible role in apoptosis [31]. Mortensen induced G2/M cell cycle arrest and caspase-dependent apoptosis of the human colon adenocarcinoma HT-29 cell line through the modulation of ERK-1/-2, AKT and NF-κB signaling [25]. In addition, terpenoids may also be involved in the Wnt/β-catenin and p38 MAPK pathways to inhibit cancer cell proliferation. A sesterterpenoid from a deep-water Alaskan sponge inhibited β-catenin response transcription (CRT) through the promotion of β-catenin degradation, which was in part implicated in anti-proliferative activity against two CRT-positive colon cancer cell lines. In short, sesterterpenoids inhibit Wnt/β-catenin signaling in colon cancer cells [32]. Siphonodictyal B, isolated from the marine sponge *Aka coralliphaga*, induced apoptosis via increased PARP cleavage, increased G1 subparts, and increased Annexin V positive cell numbers and increased ROS levels to further activate the p38 MAPK pathway [28].

### 2.4. Polysaccharides

**Figure 4:**
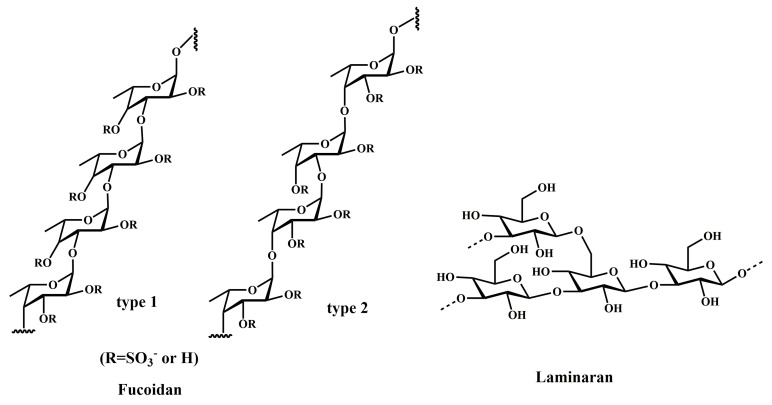
Chemical structures of marine-derived polysaccharides against CRC.

Fucoidan (Figure 4) is mainly derived from brown algae and contains fucose and sulphate groups. Owing to the complex structure of fucoidan on the one hand, the chemical composition of fucoidan produced by different species of brown algae is different, being mainly composed of L-fucose and sulphate as well as D-galactose, D-mannose, D-xylose, and uronic acid. On the other hand, the structure of fucoidan obtained from the same brown algae may be different due to diverse extraction and separation methods [33,34,35]. At present, several studies have shown that fucoidan has a variety of biological activities, including inhibiting the growth and inducing apoptosis of cancer cells [36] and inhibiting the invasion, angiogenesis, and metastasis of tumor cells [37,38,39]. In CRC cells, fucoidan can induce apoptosis through multiple pathways. Results have shown that fucoidan activates intrinsic and extrinsic apoptosis pathways through the JNK signaling pathway. At the same time, the AKT and p53 signaling pathway may also inhibit the growth of CRC through p21WAF1-mediated G1 phase cell cycle arrest [36,37,40]. In vivo studies have shown that fucoidan may prevent colon tumors by regulating the intestinal microecology and immunity in 1,2-dimethylhydrazine-induced colorectal carcinogenesis in rats. At the same time, it can activate the Hippo pathway and downregulate the β-catenin pathway to induce tumor cell apoptosis and inhibit tumor growth [41].

At the same time, fucoidan has immunostimulating effects on various types of immune cells, including macrophages and dendritic cells (DCs). Fucoidan significantly inhibits the secretion of pro-inflammatory mediators (including nitric oxide (NO) and prostaglandin E-2) and cytokines (TNF-α) in RAW 264.7 macrophages [42]. Fucoidan has been reported to promote the maturation and migration of DCs, which are antigen-presenting cells, and increase the production of IL-12 and TNF-α as well as the expression of major histocompatibility complex class I, class II, and cluster of differentiation (CD)54 and CD86 molecules. What is more, the results showed that the immune activity might be activated through toll-like receptor 4 (TLR4) and its downstream MAPK and NF-κB signaling pathways [43,44]. Finally, fucoidan can also be used as a vaccine adjuvant to increase the expression of major histocompatibility complex class II, CD25, and CD69 in spleen cells, while enhancing antigen-specific antibody production, demonstrating its use in the context of immunostimulating activity [45].

Meanwhile, it has been reported that fucoidan can play an anti-colorectal cancer role by combining multiple methods of treatment; for example, it is used in combination with radiation therapy to inhibit the proliferation and colony formation of human cancer cells, making cancer cells more sensitive to X-rays. The molecular mechanism is related to the activation of caspase protein, the inhibition of anti-apoptotic protein expression, and the enhancement of DNA fragmentation [46,47]. The use of fucoidan-coated nanoparticles to deliver anticancer drugs can improve bioavailability, and targeted radiation-induced P-selectin enhances chemoradiotherapy for CRC in mice [48,49]. Oligo-fucoidan can reduce tumor-promoting M2 macrophages in the microenvironment and act synergistically with p53 and etoposide to prevent the tumor oncogenicity of HCT-116 cells [50]. Cellular prion protein (PrPc) is thought to be involved in cell signal transduction, differentiation, survival, and cancer progression, and it has been proven that fucoidan combined with PrPc silencing had a synergistic inhibitory effect on the growth of HT-29 colon cancer cells [51].

Laminaran (Figure 4) exists in the cytoplasm of brown algae cells and is mainly composed of β-D-glucose through β-(1→3) glycosidic bond linkage [52]. The laminaran content in kelp is generally about 1%. Studies have shown that laminaran can promote the immune function of the body and protect the body from injury. Due to its low toxicity, it is often used in combination with other compounds to play an anti-colorectal cancer role. For example, the sulphated derivative of laminaran AaLs combined with linckoside L1 from the starfish induces apoptosis in vitro by deactivating AKT protein and regulating the pro-apoptotic/anti-apoptotic protein balance [53]. Laminaran and sulphated laminaran showed a synergistic effect with X-ray radiation against cancer cells, decreasing the number and size of CRC cells [54].

### 2.5. Carotenoids

**Figure 5:**
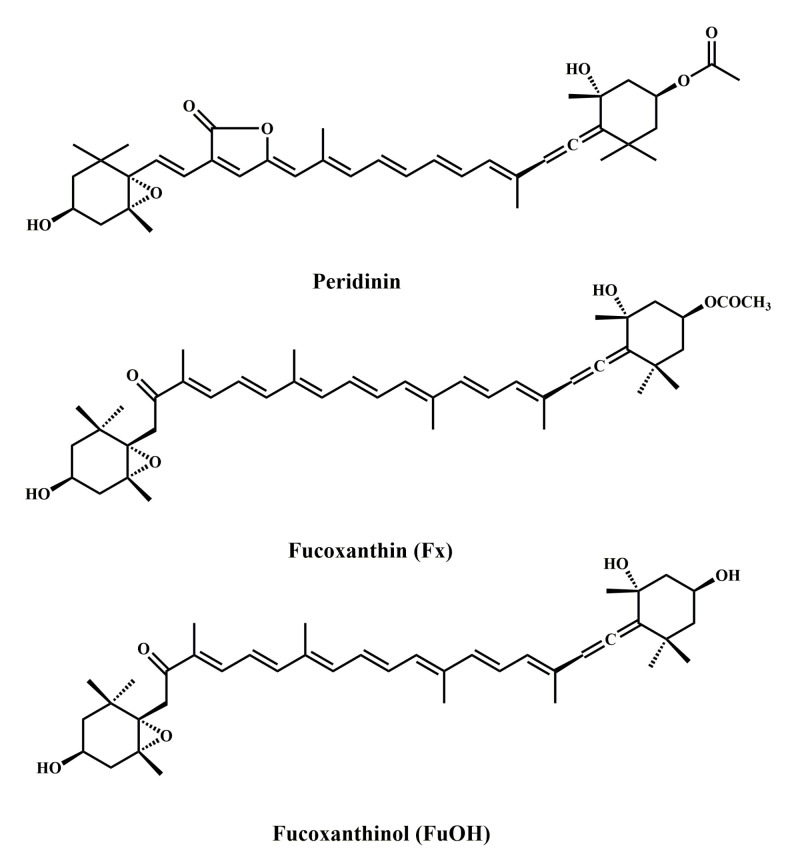
Chemical structures of marine-derived carotenoids against CRC.

Carotenoids is a general term for a class of important natural pigments that play a very important role in human health. They are widely contained in marine plants, and include peridinin (Figure 5), which is found in dinoflagellates. Studies have shown that peridinin markedly reduces the viability of DLD-1 cells in a dose-dependent manner and induces apoptosis by activating caspase-8 and caspase-9 [55].

Fucoxanthin (Fx) (Figure 5) is a carotenoid found in the chloroplasts of brown algae and has been shown to have a variety of potential bioactivity, including cancer prevention, anti-oxidation, anti-inflammation, and anti-diabetes [56,57,58,59,60,61]. These effects depend on its unique molecular structure, which includes allenic bonds and 5,6-monoepoxyde [62]. Kotake-Nara et al. showed that 5 mM Fx significantly reduced the viability of HCT-116 cells [63]. Fx induces human colon cancer cell cycle arrest in the G0/G1 phase via the up-regulation of p21 (WAF1/Cip1), which directly affects DNA replication and leads to cell apoptosis [64]. In conclusion, the anticancer mechanism of Fx involves inducing the apoptosis of CRC cells by inhibiting the G0/G1 cell cycle, caspase protein activation, MMP loss, and DNA polymerase inactivation.

Fucoxanthinol (FxOH) (Figure 5) is the main primary metabolite of Fx. The ingested Fx is hydrolyzed to FxOH by digestive enzymes such as lipase and cholesterol esterase in the gastrointestinal tract, which is then circulated throughout the body through the lymph system. Chloride intracellular channel 4 (CLIC4), a member of the CLIC family, plays an important role in the development of cancer and cell apoptosis. Studies have found that FxOH has significant anti-proliferation and apoptosis-inducing effects on the human CRC cell DLD-1, leading to an increase in anoikis-like changes. In addition, FxOH reduced the activation of CLIC4 and FAK and changed the expression and distribution of integrin β1. When the CLIC4 gene was knocked out, the apoptosis-inducing effect of FxOH almost disappeared, indicating that CLIC4 may be involved in FxOH-induced apoptosis, and FxOH can induce anoikis in CRC cells by inhibiting integrin signaling [65,66]. At the same time, FxOH weakened the epithelial–mesenchymal transition (EMT), inhibited the activation of MAPK and Stat signals, and altered metabolite profiles in CRC cells [67]. Evidence from previous studies has suggested that FxOH is a more effective apoptotic inducer than Fx. NF-κB inhibitor combined with FxOH induced apoptosis of HCT-116 cells by suppressing the inhibitor of apoptosis protein (IAP) family genes [68].

Fx and its bioconversion compound FxOH have strong anticancer effects in vitro and in vivo. Studies have reported the potential prophylactic and anticancer effects of oral Fx on azoxymethane/dextran sulphate sodium (AOM/DSS) cancer model mice. Salivary glycine is an important predictor of Fx polyp formation and tumor microenvironment attenuation in AOM/DSS mice [69]. The results showed that Fx could inhibit polyp formation, both the number and size of CRC cells were inhibited, and apoptosis-like cleaved caspase-3^high^ cells were significantly increased in both colorectal adenocarcinoma and mucosal crypts. Fx administration prominently suppressed *Bacteroidlales* and *Rikenellaceae* and increased *Lachnospiraceae* when compared to control mice. Fx also inhibited the expression and activation of integrin signaling pathway-related proteins in the mucosal tissues of AOM/DSS mice, which was consistent with the results of in vitro studies. In summary, Fx may act on CRC through multiple mechanisms. On the one hand, it may act as an anoikis activator, producing chemoprophylaxis in carcinogenic models; on the other hand, dietary Fx changes intestinal microbiota so that they play an anticancer role [70,71].

### 2.6. Other Compounds

**Figure 6:**
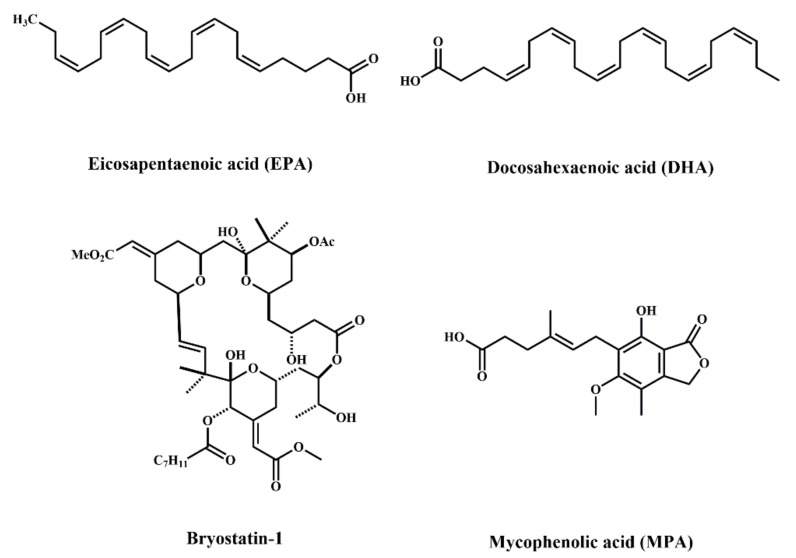
Chemical structures of other marine-derived compounds against CRC.

There are a variety of compounds of marine origin, some of which are mixtures obtained by extraction and isolation methods, which have anti-colorectal cancer effects, as shown in Table 4. In addition, polyunsaturated fatty acids (PUFAs) are essential fatty acids for human health. Omega-3 ployunsaturated fatty acids (ω-3 PUFAs) are a group of long-chain PUFAs containing eicosapentaenoic acid (EPA) (Figure 6) and docosahexaenoic acid (DHA) (Figure 6), which are abundant in marine food sources. Data from experimental studies suggest that marine ω-3 PUFAs have anti-inflammatory and anticancer biological properties [72]. ω-3 PUFAs promote the proliferation of CD4+T and CD8+T cells and the secretion of various cytokines and chemokines through immune response, thus enhancing the immune function of the body [73,74]. Han et al. showed that DHA induced the cytotoxicity of HCT-116 cells in a dose-dependent manner, mainly inducing the apoptosis of the HCT-116 cells by down-regulating survivin and Bcl-2 and up-regulating Bax, accompanied by a blockage of β-catenin complex dissociation [75]. It was demonstrated that low doses of the fish and algal oil component DHA increased the sensitivity of tumor necrosis factor-related apoptosis-inducing ligand (TRAIL) to the colon cancer cell SW620. Apoptosis induced by the combination of DHA and TRAIL was related to the mitochondrial pathway, in which Bax/Bak protein was activated, MMP was decreased, cytochrome C was released, the endoplasmic reticulum stress response was activated, and apoptosis-inhibiting protein levels were decreased [76]. Zhang et al. revealed that EPA and DHA increased the phosphorylation level of Yes-associated protein (YAP) by the GPR40/120-Gαs-PKA-MST1/2-LATS1 axis. Phosphorylated YAP migrates from the nucleus to the cytoplasm, where it remains and activates the classical Hippo-YAP pathway, playing an anticancer role in HT-29 and LoVo cells [77]. In conclusion, the therapeutic effect of ω-3 PUFAs on CRC mainly showed that it can effectively suppress the proliferation and induce apoptosis of CRC cells by inhibiting the intracellular signal transduction pathway, improving the survival rate of cancer patients.

Polyphenols are a family of bioactive substances (e.g., catechin, epigallocatechin, catechin gallate, epicatechin gallate) [78]. Studies have shown that marine polyphenols have attracted more and more attention due to their antioxidant [79,80], antibacterial, and anticancer activities [81,82]. Delgado-Roche et al. extracted and isolated a substance containing polyphenols (PF) (Table 4) from the marine angiosperm plant *Thalassia testudinum*. Crystal violet analysis showed that the survival rate of cancer cells exposed to PF was reduced in a time- and dose-dependent manner. The most sensitive cell line was HCT-15, with an IC_50_ of 36.51 ± 4.68 µg/mL and 22.47 ± 1.30 µg/mL at 24 h and 48 h, respectively. It was observed that PF induced HCT-15 cells to produce high levels of intracellular ROS. At the same time, in vivo studies have found the increased expression of Bcl-2 in PF-treated animal tumor tissues, suggesting that an intrinsic apoptotic pathway may be activated. The specific mechanism by which PF induces apoptosis needs to be studied further [83]. Subsequent studies by Hernandez-Balmaseda et al. showed that polyphenol-containing compounds (Table 4) inhibited colon cancer cell growth, motility, and angiogenesis in vitro, and promoted anti-tumor immunogenic cell death in vivo by triggering ATF4-p53-NF-κB-specific gene expression and autophagy stress pathways [84]. In conclusion, polyphenols inhibit the growth of tumor cells mainly by scavenging free radicals, inhibiting gene expression of tumor cells, and enhancing immune pathways. However, due to the complex structure and easy oxidation of polyphenols, the study of their structural characteristics and pharmacological activities is still in the primary stage.

Macrolides are a class of compounds with a carbon lactone ring in their molecular structure and have strong antibacterial action and an anticancer effect. Bryostatin-1 (Figure 6), isolated from the bryozoan *Bugula neritina*, has been studied for its anticancer activity since its discovery and identification as a macrolide in 1982 [85]. Bryostatin-1 strongly excites protein kinase C (PKC), leading to PKC activation at nanomolar concentrations, and plays an important role in regulating tumor cell growth, differentiation, invasion, metastasis, and apoptosis through PKC. Studies have shown that bryostatin-1 treatment initially increased the anti-apoptotic protein Bcl-2, but after a period of treatment, it decreased Bcl-2 through the ubiquitin degradation pathway, thus inducing the apoptosis of the tumor cells. Meanwhile, bryostatin-1 did not directly affect matrix metalloproteinases (MMPs) activity, but inhibited the expression of MMP–1, –9, –10, and –11 through PKC, thus regulating the invasion and metastasis of the tumor cells [86]. Trisciuoglio et al. isolated candidaspongiolide (CAN) macrolides from the *Candidaspongia* sp. Of marine sponges and showed that CAN induced apoptosis in a dose-dependent manner in HCT-116 CRC cells. Meanwhile, CAN activated the protein caspase-12 and induced substantial phosphorylation of eukaryotic translation initiation factor-2 (eIF2)-α in HCT-116 cells, inhibiting protein synthesis and leading to cell death [87].

AntiGan is nutrient-rich in essential amino acids, natural unsaturated fatty acids, vitamins, and minerals and was developed from the epidermis and oesophagus of the sea eel *Conger* through a non-denatured biotechnology process [88]. Lombardi et al. demonstrated that AntiGan exerts an anti-proliferative effect on SW480, Caco-2, and HT-29 cells, while the expression of apoptosis-related gene Bcl-2 was down-regulated in all of the cell lines studied [89]. According to Martinez-Iglesias et al., it increased apoptosis, decreased cell viability, and decreased the expression of COX-2 and IL-17 in HCT-116 cells, and it also enhanced the level of 5-methylcytosines (5mC) and reduced the expression of DNA methyltransferases (DNMT1 and DNMT3a). AntiGan regulates DNA methylation and histone deacetylases (SIRT) activity and expression, demonstrating that it is a novel biological anti-tumor product with epigenetic properties for the treatment of CRC [88]. Galectin can be isolated from the skin mucus of the conger eel and is involved in the development, differentiation, morphogenesis, immunity, apoptosis, and metastasis of malignant tumor cells, which may account for AntiGan’s immunomodulatory and anticancer effects [90,91,92,93]. AntiGan is composed of a variety of substances that together have biological activity. As a nutrient, it may have anticancer effects with low toxicity and side effects, providing a new direction in the development of marine nutrients.

Mycophenolic acid (MPA) (Figure 6), produced by the fermentation and metabolism of *Penicillium brevicompactum*, has anti-tumor, antiviral, immunosuppressive, and anti-inflammatory activities [94,95,96,97]. Chen et al. isolated MPA from marine-derived *Penicillium brevicompactum* OUCMDZ-4920 that showed strong cytotoxicity to HT-29, with an IC_50_ of 5.47 μmol/L [98]. Li et al. demonstrated that MPA induced apoptosis in SW620 cells through the caspase-3 pathway; the cell nucleus showed obvious pyknosis, and the cell growth cycle was arrested in the G1/S phase after the action of MPA on SW620 [99]. At present, there are few studies on the role of MPA in CRC. For one thing, MPA is catalyzed by UDP-glucuronosyl transferase to produce 7-O-glucuronic, with no biological activity in vivo, so its clinical application is limited. For another, glucuronosyltransferase may be widespread in CRC cells, and there is a certain degree of resistance to MPA [100]. Therefore, the effect of MPA on CRC needs to be studied further.

**Table 4:** Other marine-derived compounds against CRC.

Compound Name	Marine Organism	Species Name	Cell Lines	IC_50_	Mechanism	References
Polyphenolic fraction	Seagrass	*Thalassia testudinum*	HCT-15	36.51 ± 4.68 µg/mL, 24 h	Cytotoxicity; apoptosis	[83]
				22.47 ± 1.30 µg/mL, 48 h		
Thalassiolin B	Seagrass	*Thalassia testudinum*	HCT-15	51.82 ± 8.72 µg/mL, 24 h	Cytotoxicity; apoptosis	[83]
				38.75 ± 3.57 µg/mL, 48 h		
TTE	Seagrass	*Thalassia testudinum*	RKO	251.9 ± 8.8 µg/mL, 48 h 174.9 ± 8.7 µg/mL, 72 h	Anti-proliferative; block EMT; Anti-angiogenesis; ATF4-P53-NF-κB pathway	[84]
			SW480	60.5 ± 7.6 µg/mL, 48 h 58.9 ± 7.9 µg/mL, 72 h		
Candidaspongiolide (CAN)	Marine sponge	*Candidaspongia* sp.	HCT-116	≈100 nM, 48 h	Apoptosis; caspase 12 pathway	[87]
Mycophenolic acid	Marine fungi	*Penicillium brevicompactum*	HT-29	5.47 μM, \	Cytotoxicity	[98]
Neaumycin B	Marine actinomycetes	*Micromonospora*	HCT-116	3.338 μg/mL, 5 days	Cytotoxicity	[101]
PM100117	Marine actinomycetes	*Streptomyces caniferus* GUA-06-05-006A	HCT-116	3.61 μM, 72 h	Cytotoxicity	[102]
PM100118	Marine actinomycetes	*Streptomyces caniferus* GUA-06-05-006A	HT-29	4.09 μM, 72 h	Cytotoxicity	[102]
Compound 5	Marine sponge	*Theonella* sp.	DLD-1	2.50 µM, 24 h	Anti-proliferative	[103]
Compound 7	Marine sponge	*Theonella* sp.	HCT-116	0.78 µM, 24 h	Anti-proliferative	[103]
			DLD-1	0.55 µM, 24 h	Anti-proliferative	
Ganodermasides A	Marine fungi	*Pseudogymnoascus* sp. HSX2#-11	HCT-116	25 ± 1.5 µM, 24 h	Cytotoxicity	[104]
Ganodermasides B	Marine fungi	*Pseudogymnoascus* sp. HSX2#-11	HCT-116	23 ± 0.93 µM, 24 h	Cytotoxicity	[104]
Compound 1 ^1^	Marine fungi	*Penicillium oxalicum*	Caco-2	21.4 µM, 24 h	Cytotoxicity	[105]
Compound 9	Marine fungi	*Aspergillus flocculosus* 01NT-1.1.5	HCT-15	3.0 µM, 48 h	Cytotoxicity	[106]
Compound 10	Marine fungi	*Aspergillus flocculosus* 01NT-1.1.5	HCT-15	2.8 µM, 48 h	Cytotoxicity	[106]
Trichothecin	Marine fungi	*Alternaria* sp. TZP-11	HCT-116	0.25 µM, 72 h	Anti-proliferation; apoptosis; G0/G1 cell cycle arrest; block EMT; STAT3 pathway	[107]
Shellmycin A	Marine actinomycetes	*Streptomyces* sp. Shell-016	HT-29	4.69 µM, 24 h 0.85 µM, 72 h	Cytotoxicity	[108]
Shellmycin B	Marine actinomycetes	*Streptomyces* sp. Shell-016	HT-29	6.12 µM, 24 h 1.12 µM, 72 h	Cytotoxicity	[108]
Shellmycin C	Marine actinomycetes	*Streptomyces* sp. Shell-016	HT-29	13.0 µM, 24 h 4.33 µM, 72 h	Cytotoxicity	[108]
Shellmycin D	Marine actinomycetes	*Streptomyces* sp. Shell-016	HT-29	5.37 µM, 24 h 1.02 µM, 72 h	Cytotoxicity	[108]
Asperphenin A	Marine fungi	*Aspergillus* sp.	RKO	0.84 ± 0.26 µM, 72 h	Apoptosis; G2/M cell cycle arrest	[109]
Asperphenin B	Marine fungi	*Aspergillus* sp.	RKO	1.26 ± 0.43 µM, 72 h	Cytotoxicity	[109]
Cladoloside D1	Sea cucumber	*Cladolabes schmeltzii*	HT-29	16.0 ± 0.7 μM, 24 h	Cytotoxicity	[110]
Cladoloside M	Sea cucumber	*Cladolabes schmeltzii*	HT-29	14.8 ± 1.6 μM, 24 h	Cytotoxicity	[110]
Cladoloside M1	Sea cucumber	*Cladolabes schmeltzii*	HT-29	16.9 ± 0.4 μM, 24 h	Cytotoxicity	[110]
Cladoloside M2	Sea cucumber	*Cladolabes schmeltzii*	HT-29	8.5 ± 0.5 μM, 24 h	Cytotoxicity	[110]
Cladoloside N	Sea cucumber	*Cladolabes schmeltzii*	HT-29	8.8 ± 0.3 μM, 24 h	Cytotoxicity	[110]
Cladoloside Q	Sea cucumber	*Cladolabes schmeltzii*	HT-29	15.0 ± 1.4 μM, 24 h	Cytotoxicity	[110]
Compound 2	Marine actinomycetes	*Streptomyces cacaoi*	Caco-2	7.4 ± 0.3 μM, 48 h	Inhibit autophagy; induce apoptosis	[111]
Compound 1 ^1^	Marine sponge	*Aplysinella*	HCT-116	8.2 ± 0.72 µM, 72 h	Cytotoxicity	[112]
Compound 3	Marine sponge	*Aplysinella*	HCT-116	5.1 ± 0.41 µM, 72 h	Cytotoxicity	[112]
Compound 4	Marine sponge	*Aplysinella*	HCT-116	3.7 ± 0.31 µM, 72 h	Cytotoxicity	[112]
Ethanol	Seaweed	*Gracilaria verrucosa*	HCT-116	43.9 μg/mL, 48 h	Cytotoxicity	[113]
anthenosides J and K (ratio of 3:1)	Starfish	*Anthenea aspera*	HT-29	40 μM, 24 h	Apoptosis	[114]
Fraction D	Marine dinoflagellate	*Alexandrium andersoni*	HT-29	≈3 μg/mL, 48 h	Cytotoxicity; TNF pathway	[115]
Ethyl acetate	Seaweed	*Eucheuma spinosum*	HCT-116	16.82 μg/mL, 48 h	Cytotoxicity	[116]
Chloroform	Seaweed	*Eucheuma spinosum*	HCT-116	26.87 μg/mL, 48 h	Cytotoxicity	[116]
Hexane	Seaweed	*Eucheuma cottonii*	HCT-116	24.83 μg/mL, 48 h	Cytotoxicity	[116]
N-Hexane	Brown algae	*Halopteris scoparia* L. Sauvageau	Caco-2	4.53 ± 0.12 μg/mL, 48 h	Cytotoxicity; apoptosis; AKT pathway	[117]
Methanol	Brown algae	*Halopteris scoparia* L. Sauvageau	Caco-2	22 ± 0.11 μg/mL, 48 h	Cytotoxicity; apoptosis; AKT pathway	[117]
Crude extract	Marine sponge	*Latrunculia biformis*	HCT-116	4.8 µg/mL, 24 h	Cytotoxicity	[118]
			HT-29	4.0 µg/mL, 24 h		
Ethanol extract	Sea cucumber	*Holothuria atra*	WiDr	11.4 µg/mL, 24 h	Cytotoxicity	[119]
AVSC4 extract	Marine bacterium	*Bacillus flexus*	HT-29	93.4 µg/mL, 48 h	Cytotoxicity	[120]
NB extract	Nudibranch	*Dolabella auricularia*	HCT-116	1.01 ± 0.19 µg/mL, 24 h	Anti-proliferation; apoptosis; G2/M cell cycle arrest; block EMT	[121]
Crude containing liposomes	Marine sponge	*Coscinoderma* sp.	Caco-2	1.7 ± 0.18 µg/mL, 24 h	Anti-proliferative	[122]
F5	Marine plant	*Fucus vesiculosus*	Caco-2	97.4 ± 11.6 µg/mL, 48 h	Apoptosis; necrosis	[123]
			HT-29	118.8 ± 19.7 µg/mL, 48 h	Cytotoxicity	
EtOAc	Marine plant	*Fucus vesiculosus*	HT-29	170.0 ± 2.8 µg/mL, 48 h	Cytotoxicity	[123]
Hexane extract	Marine crab	*Portunus segnis*	HT-29	35.27 ± 0.71 µg/mL, 24 h 25.07 ± 0.68 µg/mL, 48 h 19.25 ± 0.22 µg/mL, 72 h	Anti-proliferative	[124]
Butanol extract	Marine crab	*Portunus segnis*	HT-29	26.63 ± 0.20 µg/mL, 24 h 15.13 ± 0.21 µg/mL, 48 h 10.12 ± 0.35 µg/mL, 72 h	Anti-proliferative; apoptosis; Caspases-3/7/9 pathway	[124]
Ethyl acetate extract	Marine crab	*Portunus segnis*	HT-29	48.14 ± 0.32 µg/mL, 24 h 34.63 ± 0.38 µg/mL, 48 h 22.86 ± 0.51 µg/mL, 72 h	Anti-proliferative	[124]
H_2_O extract	Marine crab	*Portunus segnis*	HT-29	44.33 ± 0.33 µg/mL, 24 h 31.97 ± 0.62 µg/mL, 48 h 19.38 ± 0.23 µg/mL, 72 h	Anti-proliferative	[124]

^1^ These are two different compounds, not named in the original literature.

## 3. In Vivo Study of Natural Marine Products against CRC

Due to the large difference between in vitro and in vivo environments, researchers conduct animal model experiments based on cell experiments in order to increase the persuasiveness and credibility of research results. Animal model experiments can simulate the in vivo environment and reduce the uncertainty of results. The CRC mouse model is a basic tool for studying tumor development, metastasis, and anti-tumor therapy and is divided into the carcinogenic induction model, the transplanting model, and the gene engineering model.

The long-time action of chemical reagents can generate an in situ mouse colorectal tumor. The common methods include the 1,2-dimethylhydrazine (DMH) intraperitoneal injection, N-methyl-N-nitro-nitroguanidine enema, and azoxymethane-dextran sulphate sodium (AOM-DSS) induction. The induction method of carcinogens is simple and reproducible, can be replicated in large quantities in a short time, and can simulate the carcinogenesis process to induce a well-differentiated adenocarcinoma. Methyl azo-methanol (MAM) is the active metabolite of the mutagenic agent AOM, which can enter the intestine through the bile and blood system, where it is decomposed into a methyl carbocation by intestinal flora hydrolase to alkalize DNA and induce tumor formation. DSS is a chemical inflammatory agent that causes intestinal inflammation by disrupting the intestinal mucosal barrier. Su et al. obtained β-1,3/1,6-glucan (BG136) from the marina alga *Durvillaea antarctica* and showed a reduction in the size and number of tumors in AOM/DSS-induced CRC mouse models after the administration of BG136 [125]. As described earlier in relation to carotenoids, in Section 2.5, Fx has also shown potential anticancer effects in AOM/DSS mouse models. Terasaki et al. studied Fx-related changes in the transcriptome of tumor-associated proteins in the colon mucosal tissues of AOM/DSS mice treated with or without Fx and showed that Fx produced chemoprophylaxis in AOM/DSS mice by decreasing chemokine (C-C motif) receptor 1 (CCR1) expression along with 11 cancer-related signals [126].

Tumor cell lines and patient tumor tissue were inoculated into immunodeficient mice, with these methods being referred to respectively as the cell-derived xenograft (CDX) model and the patient-derived xenograft (PDX) model. Due to the difficulty in obtaining tumor tissues from patients for the PDX model and the associated high economic cost, only the CDX model continues to be widely used in the primary stage of in vivo studies on anticancer drugs. In drug development, researchers usually perform cell proliferation tests on human tumor cell lines to detect drug inhibition and then establish CDX models using the same cell lines for preliminary validation in vivo. Chikamatsu et al. demonstrated that siphonodictyal B can induce the apoptosis of colon cancer cells in vitro by activating the ROS-p38 MAPK pathway. To demonstrate its in vivo effect, HCT-116 cells were implanted subcutaneously on the right side of mice to establish a CDX model, and the results showed that siphonodictyal B inhibited tumor growth in vivo through the p38 MAPK pathway, which was consistent with the results in vitro [28]. Bae et al. also established a CDX model and intraperitoneally injected asperphenin A three times per week for 21 days. Tumor growth was significantly inhibited in the experimental group compared with the control group, and the inhibition rate was 68.7 ± 17.1% [109]. As shown in Table 5, the CDX model was used in most in vivo experiments thanks to the simplicity of the method, short time period required for tumor formation, and high tumor formation rate.

Gene engineering models use transgenic technology to directly transfer tumor-forming-related genes into animals for expression, so that the carcinogenic process and pathological manifestations of tumor models can be made to be similar to those of the human body. The adenomatous polyposis coli (APC) gene, as a tumor suppressor gene, directly participates in the Wnt signaling pathway and regulates β-catenin expression in order to regulate cell proliferation. APC mutation results in the growth disorder of intestinal epithelial cells, leading to the formation of CRC, and is an early event of colorectal tumors [127]. Thus, targeting the APC gene is helpful in the prevention and treatment of colorectal tumors, and APC^Min/+^ transgenic mice are the classic model for studying the occurrence and development of intestinal tumors [128,129]. The Kras^V12G^/APC^+/1638 N^ transgenic mouse model and APC^Min/+^ Tp^53-/-^ double gene mutation mouse model are also used by some scholars in the study of CRC [130,131]. The pathogenesis and etiology of CRC are very complex and the in vivo study of CRC are an indispensable aspect of basic research and development. Only by selecting appropriate mouse models, according to experimental purposes, can we more accurately clarify the mechanism of action of marine drugs on CRC.

**Table 5:** Marine compounds studied in vivo against CRC.

Compound Name	Marine Organism	Species Name	Cell Lines	Mode of Tumor Formation	Delivery Way	Doses	Tumor Suppressor	References
Ohmyungsamycin A	Marine actinomycetes	*Streptomyces* sp.	HCT-116	Injected subcutaneously into the flanks of the mice	Intraperitoneal injection	10 mg/kg; Three times per week	Tumor inhibition rate: 52.1%	[18]
Peptide, (P6)	Bvalve mollusk	*Arca inflata*	HT-29	Injected subcutaneously into the left armpit of mice	Intraperitoneal injection	30 mg/kg; Every day	Tumor inhibition rate: 72.66%	[22]
Siphonodictyal B	Marine sponge	*Aka coralliphaga*	HCT-116	Implanted subcutaneously into the right flanks of mice	Intraperitoneal injection	20 mg/kg; Every 3 days	Tumor growth inhibition	[28]
Polyphenolic fraction	Seagrass	*Thalassia testudinum*	HCT-15	Injected subcutaneously into the lower right flank region of mice	Oral gavage	25 mg/kg; Three days a week	Tumor growth inhibition	[83]
TTE	Seagrass	*Thalassia testudinum*	CT-26	Injected subcutaneously into the right dorsal side of mice	Oral gavage	100 mg/kg; Every day	Tumor inhibition rate: 69.39 ± 6.7%	[84]
Caulerpin	Green algae	*Caulerpa cylindracea*	SW480	Injected subcutaneously into the right flanks of mice	Oral gavage	30 mg/kg; Every other day	Tumor growth inhibition	[12]
Asperphenin A	Marine fungi	*Aspergillus* sp.	RKO	Injected subcutaneously into the flanks of the mice	Intraperitoneal injection	8 mg/kg; Three times per week	Tumor inhibition rate: 68.7 ± 17.1%	[107]
SPS-CF	Green alga	*Capsosiphon fulvescens*	HT-29	Injected subcutaneously into the back of mice	Intraperitoneal injection	400 mg/kg/day	Tumor inhibition rate: 20%	[131]
Fucoxanthin (Fx)	Brown algae	*\*	HT-29	Injected subcutaneously into the right femoral region of mice	Oral gavage	2.5 mg/kg; Every 2 or 3 days	Tumor growth inhibition	[132]

## 4. Clinical Study of Natural Marine Compounds

Since the first marine-derived compound, ara-C (CytosarU), was authorized by the FDA for the clinical treatment of leukaemia in 1969, more and more marine drugs have been approved for the treatment of cancer. As of April 2022, 11 anticancer drugs from marine compounds have been approved, as shown in Table 6 [5]. Among the later approved drugs, antibody-drug conjugates (ADCs) have become a milestone in targeted therapy, with the antibodies of the ADC, which selectively target antigen-positive cancer cells, delivering cytotoxic agents into malignant cells without harming normal cells [133]. Currently, most marine anticancer drugs or potential anticancer drugs are ADCs, which illustrates their importance in cancer treatment. Marine resources are playing an increasingly important role as a natural treasure trove for anticancer drugs, with mollusks and cyanobacteria being the main sources for finding such anticancer compounds.

A large number of marine drugs are also entering clinical trials, as shown in Table 7, offering the hope of finding promising anticancer drugs. Currently, the standard treatment options for CRC include surgical resection and radiotherapy. For example, malignant colorectal polyps are resected endoscopically or by segmental resection, while tumors with high metastatic potential are treated with surgical resection plus adjuvant chemotherapy, immunotherapy, or radiation therapy to inhibit cancer metastasis. However, these treatment modalities are prone to tumor recurrence, drug resistance, adverse events, and poor prognosis, with low survival rates [1]. Plocabulin is a novel microtubule-disrupting anti-tumor agent of marine origin that is currently undergoing phase II clinical trials in advanced or metastatic CRC after standard treatment. Some studies have shown that the novel anticancer agent derived from globulin of marine origin has potent cytotoxic activity in human tumor organoids derived from CRC patients; in cultures of tumor organoids derived from three therapy-naive individuals, globulin was more cytotoxic than SN38, the active derivative of irinotecan, which is a drug widely used to treat CRC [134]. In addition, the clinical benefit rate (*n* = four of six evaluable patients) observed among CRC patients treated with plocabulin was remarkable, as all six patients had received prior standard treatment with oxaliplatin, fluorouracil, irinotecan, and bevacizumab and three had received investigational drugs. Despite this heavy pretreatment, globulin improved the clinical benefit achieved with the last prior therapy line in two of four patients. In agreement with the radiological disease stabilization, the tumor marker CEA decreased in all three CRC patients who were analyzed [135]. Plocabulin targets the tubulin dimers at a new binding site and causes apoptosis by inhibiting tubulin polymerization. Moreover, globulin is also reported to inhibit angiogenesis in endothelial cells. Therefore, plocabulin has the potential to become a clinical agent for the treatment of CRC.

Compounds of marine food origin could be a good option for CRC prevention and improved survival, as shown in Table 8. Fx and FxOH are anticancer agents which are contained and are abundant in edible brown algae. According to previous studies, dietary seaweed containing Fx or FxOH has a positive benefit as a chemopreventitive and/or chemotherapeutic agent for those at risk of CRC [136]. In addition, marine ω-3 PUFAs, primarily found in dark fish, may prevent CRC progression, in part through the inhibition of prostaglandin-endoperoxide synthase 2 (PTGS2) [137]. Numerous clinical study data have shown that marine ω-3 fatty acid is preferably associated with a lower risk of microsatellite instability (MSI) tumors, and together suggest that marine ω-3 may improve the survival of colon cancer patients by modulating the unique microenvironment in tumors that lack KRAS mutation and arise from the MSI pathway [138].

In conclusion, clinical studies have shown the potential of compounds of marine origin against CRC.

## 5. Discussion

In this review, by summarizing the source, structure, and in vitro pharmacological activities and mechanisms of marine compounds, we have attempted to find the connections between them and to provide a basis for the marine drug treatment of CRC. The first connection is the relationship between structure and source. Alkaloids are mainly derived from cyanobacteria with a nitrogen fixation ability, fungi that are capable of symbiotic relationships, and sponges and peptides are mainly derived from bacteria, periphyton, mollusks, and sponges. Meanwhile, terpenoids are mainly derived from sponges, corals, and brown algae, polysaccharides are mainly derived from brown algae and marine animals, such as sea cucumbers, and carotenoids are mainly from marine plants, such as brown algae. The second connection is the relationship between structure and pharmacological activity. The potency of diketopiperazine alkaloids may be due to sulphide groups, as in the case of GQQ-792, reduced-gliotoxins, and plinabulin, which are both diketopiperazine compounds [5,9,15]. Sulphide groups form mixed disulphides with proteins or antioxidants (e.g., glutathione) and thus have antioxidant effects. Differences in the nitrogen-containing groups of alkaloids are also the main reason for differences in the potency of the compounds; for example, hemimycalin E is more cytotoxic than hemimycalin C and hemimycalin D, which have similar structures [10]. In addition, alkaloids inhibit cell proliferation and regulate programmed cell death (e.g., apoptosis), and even inhibit cell migration and invasion, mainly by targeting topoisomerases and microtubule protein aggregation. More than 90% of the peptides trigger apoptosis through targeted apoptotic mechanisms, such as the mitochondrial and death receptor pathways. Both apoptotic pathways require the mitochondria-mediated activation of caspases. Activation of the mitochondrial apoptotic pathway is mainly due to factors such as intracellular ROS production and Ca^2+^ overload. When Ca^2+^ homeostasis is disrupted in tumor cells, high levels of ROS are generated, which activates apoptosis. Sesterterpenoid inhibits Wnt/β-catenin signaling in colon cancer cells [23]. The mechanisms by which polysaccharides, carotenoids, and polyunsaturated fatty acids exert their anticancer effects are weak compared to other compounds; however, they can exert potent anticancer effects when combined with other tools, e.g., the inhibition of colonization of colon cancer cells in combination with radiation therapy.

In the basic research on the effects of marine drugs on CRC, in vitro experiments dominate the field, but the biological process of CRC is extremely complex and is regulated by many factors. Since the CRC model induced by AOM/DSS simulates the whole process from normal mucosa to inflammation and then to tumor formation, it can truly reflect the influence of intestinal microbes, diet, and drugs on colorectal tumors. Marine polysaccharides and carotenoids are mostly present in brown algae and have anti-inflammatory and anti-tumor biological effects. Most of these compounds were orally administered in the AOM/DSS mouse model, and the results showed that they had certain inhibitory effects on tumor formation and low toxicity to the liver and kidney. In contrast, marine polypeptides (e.g., peptides) and terpenoids (e.g., siphonodictyal B) are mostly administered by injection in the CDX model due to their structural properties and have been shown to have a significant inhibitory effect on tumor size relative to the control group [22,28]. Although the carcinogenic induction model and the CDX model are widely used in CRC in vivo studies, they have their disadvantages. There are many problems in the carcinogen-induced CRC model, such as large individual differences, the difficulty of observing the tumor size, and indirect hepatotoxicity caused by carcinogens. The tumor microenvironment is not realistic, and metastasis rarely occurs in CDX models. Therefore, for in vivo studies of marine drugs on CRC, multiple models should be used for repeated validation to effectively evaluate their mechanism in vivo.

In clinical research, the approval and application of ADC applications is a milestone in the search for targeted anticancer drugs from marine compounds [133]. Antibody-drug coupling can selectively target antigen-positive cancer cells, delivering cytotoxic drugs to malignant cells without harming normal cells. As of April 2022, there were 11 FDA-approved anticancer compounds of marine origin and 23 marine anticancer drugs in clinical trials, of which six are related to the treatment of CRC by targeting HER2, 11 by microtubules, 1 by BCMA, and 1 by EGFR. This shows that marine drugs offer the great possibility of finding a treatment for CRC by targeting HER2 [5]. Furthermore, plocabulin is a novel microtubule-disrupting anti-tumor agent of marine origin that is currently undergoing phase II clinical trials in advanced or metastatic CRC after standard treatment and has the potential to become a clinical agent for the treatment of CRC. Finally, people may be able to prevent CRC by consuming brown algae containing Fx and fish containing ω-3 PUFAs.

## 6. Conclusions

**Figure 7:**
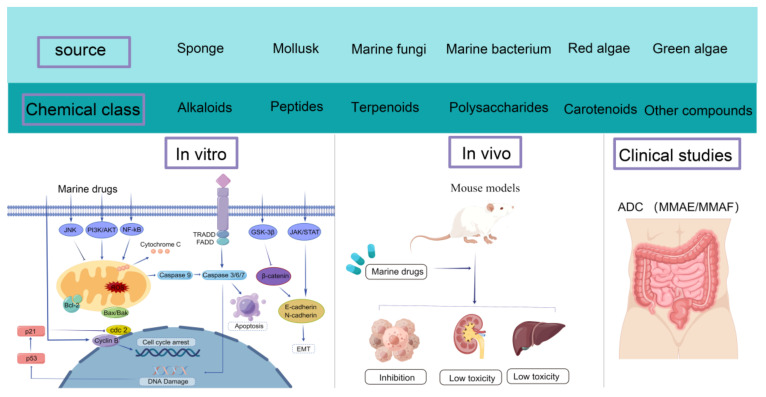
In vitro, in vivo, and clinical studies of marine compounds against colorectal cancer. By Figdraw (www.figdraw.com, accessed on 24 May 2022).

From in vitro studies, this review has summarized the marine compounds with anti-colorectal cancer effects from different sources based on their chemical structures, such as alkaloids, peptides, terpenoids, polysaccharides, carotenoids, etc. We have also elucidated the main pharmacological mechanisms of marine compounds against CRC, which include the inhibiting of the angiogenesis, invasion, and metastasis of cancer cells by inhibiting intracellular signal transduction, influencing cyclin to induce cell cycle arrest, activating caspase protein, and inactivating DNA polymerase. In this paper, the effects of marine drugs on colorectal cancer were reviewed based on reported in vivo experimental results in model mice. In terms of clinical research, this paper has systematically summarized the marine anticancer compounds that are currently approved and in the process of approval and has summarized the sources and targets of action of these compounds. The main contents of this paper are shown in Figure 7. Finally, the prospects for the clinical application and development of marine compounds were outlined. This will accelerate the exploitation of marine resources with a view to the early eradication of CRC.

## Figures and Tables

**Table 1:** Marine-derived alkaloids against CRC.

Compound Name	Marine Organism	Species Name	Cell Lines	IC_50_	Mechanism	References
Brevianamide C	Marine fungi	*Penicillium brevicompactum*	HCT-116	15.6 µM, 72 h	Anti-proliferation	[8]
Hemimycalin C	Red Sea sponge	*Hemimycale* sp.	HCT-116	18.6 ± 0.12 µM, 72 h	Anti-proliferation	[10]
Hemimycalin D	Red Sea sponge	*Hemimycale* sp.	HCT-116	17.1 ± 0.09 µM, 72 h	Anti-proliferation	[10]
Hemimycalin E	Red Sea sponge	*Hemimycale* sp.	HCT-116	8.6 ± 0.06 µM, 72 h	Anti-proliferation	[10]
Manzamine A	Marine sponge	*Haliclona* sp.	HCT-116	4.5 ± 1.7 µM, 24 h	Anti-proliferation; apoptosis; G0/G1 cell cycle arrest; block EMT	[11,16]
Reduced-gliotoxin	Marine fungi	*Neosartorya pseudofischeri*	HCT-116	≈5 µM, 24 h	Anti-proliferation; apoptosis; anoikis	[9]
			HT-29	≈7 µM, 24 h	Anti-proliferation; apoptosis; anoikis	
GQQ-792	Mangrove endophytic fungi	*Tilachlidium* sp.	HCT-116	≈1.19 µM, 72 h	Anti-proliferation	[15]
Caulerpin	Green algae	*Caulerpa cylindracea*	LoVo	20 µM, 48 h	Anti-proliferation Apoptosis; AMPK/mTOR pathway	[12]

**Table 2:** Marine-derived peptides against CRC.

Compound Name	Marine Organism	Species Name	Cell Lines	IC_50_	Mechanism	References
L-Glutaminase	Marine bacterium	*Halomonas meridian*	LS-174-T	7 µg/mL, 72 h	Anti-proliferation; apoptosis	[17]
			HCT-116	13.2 µg/mL, 72 h	Anti-proliferation; apoptosis	
Ohmyungsamycin A	Marine actinomycetes	*Streptomyces* sp.	HCT-116	7.61 µM, 72 h	Anti-proliferation; apoptosis; G0/G1 cell cycle arrest	[18]
Actinomycin V	Marine actinomycetes	*Streptomyces* sp.	HCT-116	2.85 ± 0.10 nmol/L, 48 h	anti-proliferation; apoptosis; PI3K/AKT pathway	[19]
			HT-29	6.38 ± 0.46 nmol/L, 48 h	Anti-proliferation	
			SW620	6.43 ± 0.16 nmol/L, 48 h	Anti-proliferation	
			SW480	8.65 ± 0.31 nmol/L, 48 h	Anti-proliferation	
Androsamide	Marine actinomycetes	*Nocardiopsis* sp.	Caco-2	13 µM, 48 h	Anti-proliferation; block EMT	[20]
			HCT-116	21 µM, 48 h	Anti-proliferation	
Laxaphycin B4	Marine cyanobacterium	*Hormothamnionen teromorphoides*	HCT-116	1.7 µM, 48 h	Anti-proliferation	[21]
Laxaphycin A2	Marine cyanobacterium	*Hormothamnionen teromorphoides*	HCT-116	23 µM, 48 h	Anti-proliferation	[21]
Peptide, (P6)	Bvalve mollusk	*Arca inflata*	DLD-1	2.14 ± 0.28 µg/mL, 48 h	Anti-proliferation; apoptosis; S/G2 cell cycle arrest; p38 MAPK pathway	[22]
			HT-29	4.43 ± 0.15 µg/mL, 48 h	Anti-proliferation	
			HCT-116	10.88 ± 0.72 µg/mL, 48 h	Anti-proliferation	
Catfish muscle	Marine catfish	*Tachysaurus dussumieri*	HT-29	20 µg/mL, 24 h	Anti-proliferation	[24]

**Table 3:** Marine-derived terpenes against CRC.

Compound Name	Marine Organism	Species Name	Cell Lines	IC_50_	Mechanism	References
Trichodermaloids A	Marine sponge symbiotic fungi	*Dysidea* sp. and *Trichoderma* sp.	SW620	9.3 ± 2.2 µM, \	Anti-proliferation	[26]
Trichodermaloids B	Marine sponge symbiotic fungi	*Dysidea* sp. and *Trichoderma* sp.	SW620	8.6 ± 1.9 µM, \	Anti-proliferation	[26]
Trichodermaloids C	Marine sponge symbiotic fungi	*Dysidea* sp. and *Trichoderma* sp.	SW620	12.7 ± 0.6 µM, \	Anti-proliferation	[26]
Rhinomilisin E	Marine sponge symbiotic fungi	*Dysidea* sp. and *Trichoderma* sp.	SW620	22.7 ± 2.3 µM, \	Anti-proliferation	[26]
Smenospongine	Marine sponge	*Haliclona* sp.	HCT-116	8 µM, 72 h	Anti-proliferation; apoptosis; G2/M and G1 cell cycle arrest; DNA damage	[27]
			RKO	15 µM, 72 h	Anti-proliferation	
			HT-29	10 µM, 72 h	Anti-proliferation; apoptosis; G1 cell cycle arrest	
Ilimaquinone	Marine sponge	*Verongula rigida*	HT-29	13 µM, 72 h	Anti-proliferation; apoptosis; G1 cell cycle arrest; DNA damage	[27]
Dactylospontriol	Marine sponge	*Verongula rigida*	HCT-116	19 µM, 72 h	Anti-proliferation; G1 cell cycle arrest	[27]
Siphonodictyal B	Marine sponge	*Aka coralliphaga*	HCT-116	1 µM, 24 h	Apoptosis; G1 cell cycle arrest; PI3K inhibitor; p38 MAPK pathway	[28]
13-Acetoxysarcocrassolide	Alcyonacean coral	*Lobophytum crassum*	HCT-116	1.36 ± 0.27 µg/mL, 72 h	Anti-proliferation	[29]
			LoVo	1.38 ± 0.37 µg/mL, 72 h	Anti-proliferation	
			DLD-1	1.64 ± 0.36 µg/mL, 72 h	Anti-proliferation	
14-O-acetylsarcophytol B	Marine soft coral	*Klyxum flaccidum*	DLD-1	11.7 ± 4.8 µg/mL, \	Cytotoxicity	[30]
Flaccidenol A	Marine soft coral	*Klyxum flaccidum*	DLD-1	6.0 ± 0.4 µg/mL, \	Cytotoxicity	[30]
Mertensene	Red alga	*Pterocladiella capillacea*	HT-29	56.50 ± 8.68 µg/mL, 72 h	Anti-proliferation; apoptosis; G2/M cell cycle arrest; ERK-1/-2, AKT and NF-κB activation	[25]
			LS-174-T	49.77 ± 4.51 µg/mL, 72 h	Anti-proliferation	
Sipholenol A	Red Sea sponge	*Callyspongia siphonella*	HCT-116	14.8 ± 2.33 µM, 72 h	Anti-proliferation; apoptosis; G2/M and S cell cycle arrest	[31]
Sipholenol L	Red Sea sponge	*Callyspongia siphonella*	HCT-116	19.8 ± 3.78 µM, 72 h	Anti-proliferation; apoptosis; G2/M and S cell cycle arrest	[31]
Sesterterpenoid	Marine sponge	*Monanchora pulchra*	HCT-116	43.5 µM, 48 h	Anti-proliferation; Wnt/β-Catenin pathway	[32]

**Table 6:** FDA-approved natural marine compounds with anticancer activity.

Compound Name	Marine Organism	Chemical Class	Molecular Target	Cancer Type	Year of FDA-Approval
Crytarabine	Marine sponge	Nucleoside	DNA polymerase	Leukemia	1969
Eribulin mesylate	Marine sponge	Macrolide	Microtubules	Metastatic breast cancer	2010
Brentuximab vedotin	Mollusk/ cyanobacterium	ADC (MMAE) ^1^	CD30 and microtubules	Anaplastic large T-cell systemic malignant lymphoma, Hodgkin disease	2011
Trabectedin	Tunicate	Alkaloid	Minor groove of DNA	Soft tissue sarcoma and ovarian cancer	2015
Panobinostat	Marine sponge	Hydroxamic acid	Histone	Multiple myeloma	2015
Plitidepsin	Tunicate	Dipsipetide	eEF1A2	Multiple myeloma, leukemia, lymphoma	2018 (Australia) ^2^
Polatuzumabvedotin	Mollusk/ cyanobacterium	ADC (MMAF) ^1^	CD76b and microtubules	Non-Hodgkin lymphoma, chronic lymphocytic leukemia, lymphoma, B-cell lymphoma, folicular	2019
Enfortumabvedotin	Mollusk/ cyanobacterium	ADC (MMAE)	Nectin-4	Metastatic urothelial cancer	2019
Belantamabmafodotin	Mollusk/ cyanobacterium	ADC (MMAF)	BCMA	Relapsed/refractory multiple myeloma	2020
Lurbinectedin	Tunicate	Alkaloid	RNA polymerase II	Metastatic small-cell lung cancer	2020; 2021 (Australia)
Disitamab vedotin	Mollusk/ cyanobacterium	ADC (MMAE)	HER2	Urothelial carcinoma advanced cancer, gastric cancer, HER2 overexpressing gastric carcinoma, advanced breast cancer, solid tumors	2021

^1^ MMAE and MMAF, monomethyl auristatin E and F, are microtubule-associated inhibitors. ^2^ Time for approval by the Australian regulatory authorities.

**Table 7:** Clinical trials of anticancer natural marine compounds.

Compound Name	Marine Organism	Chemical Class	Molecular Target	Cancer Type	Clinical phase
Plinabulin	Marine fungi	Diketopiperazine	Microtubules	Non-small-cell lung cancer, Brain tumor	Phase III
Marizomib	Marine bacterium	Bata-lactone-gamma lactam	20S proteasome	Non-small-cell lung cancer, Pancreatic cancer, Melanoma, Lymphoma, Multiple myeloma	Phase III
Plocabulin (PM184)	Marine sponge	Polyketide	Microtubule	Solid tumors	Phase II
Tisotumab vedotin	Mollusk/ cyanobacterium	ADC (MMAE)	Tissue factor and microtubules	Ovary cancer, Cervix cancer, Endometrium cancer, Bladder cancer, Prostate cancer, Head and neck cancer, Esophagus cancer, Lung cancer	Phase II
Ladiratuzumabvedotin (SGNLIV1A)	Mollusk/ cyanobacterium	ADC (MMAE)	LIV-1 and microtubules	Breast cancer	Phase II
Telisotuzumabvedotin (ABBV-399)	Mollusk/ cyanobacterium	ADC (MMAE)	c-Met	Solid tumors	Phase II
CAB-ROR2 (BA-3021)	Mollusk/ cyanobacterium	ADC (MMAE)	ROR2	Solid tumor, non-small-cell lung cancer, triple-negative breast cancer, soft tissue sarcoma	Phase II
CX-2029 (ABBV-2029)	Mollusk/ cyanobacterium	ADC (MMAE)	CD71	Solid tumor, head and neck cancer, non-small-cell lung, pancreatic cancer, diffuse large B-cell lymphoma	Phase II
W0101	Mollusk/ cyanobacterium	ADC (MMAE)	IGF-R1	Advanced or metastatic solid tumors	Phase II
ARX-788	Mollusk/ cyanobacterium	Amberstatin269	HER2 and microtubules	Breast cancer, gastric cancer	Phase I
XMT-1536	Mollusk/ cyanobacterium	ADC (Dolaflexin)	NaPi2b and microtubules	Solid tumors	Phase I
ALT-P7	Mollusk/ cyanobacterium	ADC (MMAE)	HER2 and microtubules	Breast cancer, gastric cancer	Phase I
MORAb-202	Marine sponge	ADC (Macrolide)	Microtubules	Solid tumors	Phase I
PF-06804103	Mollusk/ cyanobacterium	ADC (Auristatin variant)	HER2	Breast neoplasms, stomach neoplasms, esophagogastric junction neoplasm, carcinoma, Non-small-cell lung	Phase I
ZW-49	Mollusk/ cyanobacterium	ADC (Auristatin variant)	HER2	HER2-expressing cancers	Phase I
MRG003	Mollusk/ cyanobacterium	ADC (MMAE)	EGFR	Non-small-cell lung	Phase I
STRO-002	Marine sponge	Taltobulin	Folate receptor alpha (FolRa)	Ovarian, endometrial	Phase I
RC-88	Mollusk/ cyanobacterium	ADC (MMAE)	Mesothelin	Solid tumors	Phase I
SGN-B6A	Mollusk/ cyanobacterium	ADC (MMAE)	Integrin beta-6	Solid tumors	Phase I
SGN-CD228A	Mollusk/ cyanobacterium	ADC (MMAE)	CD228	Solid tumors	Phase I
FOR-46	Mollusk/ cyanobacterium	ADC (MMAF)	CD46	Multiple myeloma, prostate	Phase I
A-166	Mollusk/ cyanobacterium	Duostatin 5	HER2	HER2-expressing cancers	Phase I
Cofetuzumabpelidotin (ABBV-647)	Mollusk/ cyanobacterium	ADC (Auristatin variant)	PTK7	Non-small-cell lung	Phase I

**Table 8:** Edible natural marine product that has preventive effects on CRC.

Compound Name	Marine Organism	Chemical Class	Effect on Colorectal Cancer	Phase	References
Fucoxanthin\fucoxanthinol	Brown algae	Carotenoid	Prevention of colorectal cancer	\	[132]
Marine omega-3 fatty acid	Marine fish	Polyunsaturated fatty acids	Prevention and improve colon cancer survival	NCCTG Phase III	[131,139,133]
